# Selenium and Health: An Update on the Situation in the Middle East and North Africa

**DOI:** 10.3390/nu11071457

**Published:** 2019-06-27

**Authors:** Sohayla A. Z. Ibrahim, Abdelhamid Kerkadi, Abdelali Agouni

**Affiliations:** 1 Department of Pharmaceutical Sciences, College of Pharmacy, QU health, Qatar University, P.O. Box 2713, Doha, Qatar; 2 Department of Nutrition, College of Health Sciences, QU health, Qatar University, P.O. Box 2713, Doha, Qatar

**Keywords:** selenium, chronic diseases, Middle East, North Africa

## Abstract

Selenium (Se) is an important trace element that should be present in the diet of all age groups to provide an adequate intake. Se is incorporated in 25 known selenoproteins, which mediate the biological effects of Se including, immune response regulation, maintenance of thyroid function, antioxidant defense, and anti-inflammatory actions. A balanced intake of Se is critical to achieve health benefits because depending on its status, Se has been found to play physiological roles or contribute to the pathophysiology of various diseases including, neurodegenerative diseases, diabetes, cancer, and cardiovascular disorders. Se status and intake are very important to be known for a specific population as the levels of Se are highly variable among different populations and regions. In the Middle East and North African (MENA) region, very little is known about the status of Se. Studies available show that Se status is widely variable with some countries being deficient, some over sufficient, and some sufficient. This variability was apparent even within the same country between regions. In this review, we summarized the key roles of Se in health and disease and discussed the available data on Se status and intake among countries of the MENA region.

## 1. Introduction

Selenium (Se) is a semi-solid metal that was discovered in 1817 as a byproduct of sulphuric acid synthesis. It belongs to group 6 of the periodic table with an atomic number of 34. It is a red colored powder; however, in vitreous form it is observed as black, and in crystalline form it is observed as metallic gray. Se exists in many different oxidation states including 2+, 4+, 6+, and 2- [1]. Se is present in small amounts in food mostly in organic forms (selenomethionine and selenocysteine) and rarely in inorganic forms (selenate and selenite) [2]. Selenomethionine is usually derived from animal sources in addition to cereal products grown in areas where the soil is rich in Se whereas selenocysteine is only obtained from animal sources [3]. Inorganic forms are the major sources of Se incorporated into dietary supplements [2]. Se concentrations in the soil where plants are grown or where animals are raised are important indicators of Se intake in a country leading to tremendous variations among different countries in Se status [2,4]. The most common sources of Se are Brazil nuts, cereals, offal, fish, eggs, poultry, and vegetables [3,4,5].

Se is a necessary trace element that should be present in the diet of all age groups. The Recommended Dietary Allowance (RDA) of Se is generally based on the optimum amount that can maximize the activity of selenoprotein glutathione peroxidase [2,3,4]. This value was estimated to be 55 µg/day for both males and females [2,3,4,6]. The Estimated Average Requirement (EAR) was found to be around 45 µg/day for 19–50-year-old men and women and the tolerable upper intake level (UL) is around 400 µg/day [3,4]. However, the optimal amount required to achieve maximum health benefits is unknown [3]. The World Health Organization (WHO) recommends that the optimum serum concentration of Se for healthy adults is 39.5–194.5 ng/ml and that the concentration that maximizes glutathione peroxidase activity is between 70 and 90 ng/ml [7,8]. 

Very limited data exist on Se toxicity in humans; however, animal studies are available on chronic Se poisoning [4]. It was reported that an intake of around 800 μg/day of Se would cause no observed adverse effect level (NOAEL), 1,540 to 1,600 μg/day of Se would result in low observed adverse effect level (LOAEL) and 5,000 μg/day of Se is the toxic level where selenosis is expected to happen [3]. LOAEL is associated with symptoms of hair and nail brittleness and loss, garlic like breath odor, gastrointestinal disturbances, and fatigue [3,4]. Selenosis can be associated with serious respiratory, renal, and cardiac complications [3]. 

Se status can be assessed through blood, urine, nails, and hair levels. Blood specimens can be either whole blood, erythrocytes, serum, or plasma. Erythrocytes, hair, and nails reflect long-term status opposing to plasma or serum which reflect short-term levels. It is challenging to compare Se levels across different countries due to the wide variations in methodologies of Se detection among different laboratories [9,10].

## 2. Selenium: A Focus on Biology 

### 2.1. Selenoproteins as Mediators of Se Actions

Se is an essential nutrient to human biology. The beneficial roles of Se to human health and its requirement for life have been known for several decades. Se exists in 25 known selenoproteins, some of which have important known biological functions [11]. The critical role of Se in human health is particularly underscored by being the sole trace element specified in the genetic code as selenocysteine, today recognized as the 21st amino acid, which has its proper genetic code, specific biosynthesis pathway, and insertion mechanism. This amino acid exists in the active site of many selenium-dependent enzymes that are responsible for multiple essential functions in the human body [1,12,13]. In this case, Se acts as a redox center. For example, thioredoxin reductase, a selenoenzyme, participates in DNA synthesis by reducing nucleotides which then helps in controlling the redox state intracellularly [3]. Another example is iodothyronine deiodinases which activates thyroid hormone [1]. Other selenoproteins have different functions including anti-inflammatory and antioxidant effects; however, some of those functions are yet to be identified. The biological effects of Se also include its participation in immune responses and thyroid function whereas its serum level can also be linked with risk of diabetes, cardiovascular disease (CVD), and cancer [5]. Table 1 summarizes the main functions and significance of the different known selenoproteins.

### 2.2. Synthesis and Co-Translational Incorporation of Selenocysteine in Humans

**Figure 1:**
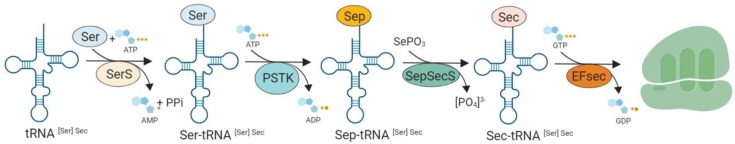
Synthesis and co-translational incorporation of selenocysteine into selenoproteins in humans.

The cycle of selenocysteine synthesis and incorporation in humans begins with the attachment of L-serine to non-cognate tRNA^Sec^ by seryl-tRNA synthetase, an error that is not edited. This seryl group gets phosphorylated by O-phosphoseryl-tRNA^Sec^ kinase (PSTK) resulting in phosphoseryl (Sep)-tRNA^Sec^ intermediate. Finally, this Sep-tRNA^Sec^ is converted into selenocysteinyl (Sec)-tRNA^Sec^ through O-phosphoseryl-tRNA^Sec^:selenocysteinyl-tRNA^Sec^ synthase (SepSecS) in the presence of selenophosphate and pyridoxal phosphate. Selenophosphate is the main Se donor in humans synthesized from adenosine triphosphate (ATP) and selenide, the products of selenocysteine degradation, by selenophosphate synthetase (SPS2). A specialized elongation factor (EFsec) then delivers Sec-tRNA^Sec^ to the ribosome. SElenoCysteine Insertion Sequence (SECIS), located in the 3′-UTR, helps localize EFsec and Sec-tRNA^Sec^ near the translation site and forms a stem loop structure which is necessary for decoding of selenocysteine UGA codon and its insertion into the nascent protein [14,15,16,17]. The cycle of selenocysteine synthesis and incorporation into selenoproteins in humans is summarized in Figure 1.

## 3. Selenium in Health and Disease: The Importance of a Balanced Intake

Se depending on its status has been reported to play crucial roles in normal physiology or contribute the pathophysiology of various diseases. Se has antioxidant and anti-inflammatory actions and therefore various studies have assessed the impact of Se status on conditions characterized by inflammation and oxidative stress including, neurodegenerative diseases, diabetes, cancer, and cardiovascular disorders [18].

### 3.1. Pregnancy

During pregnancy, the demand for multiple nutrients increase owing to many physiological changes, resulting in deficiencies of many vitamins and minerals [9,19]. Those deficiencies might sometimes lead to complications to the fetus or to the mother. Se levels were found to be significantly reduced during pregnancy [9,19]. During the first trimester, GPx activity was found to be reduced significantly and this low level was maintained throughout the second and third trimesters with a slight increase during delivery [20]. Subsequently, in the second and third trimesters, Se levels were noted to be significantly reduced with further reduction during delivery [20]. Due to this increased demand, the RDA for Se during pregnancy was increased from 55 μg/day to 60 μg/day [19,21]. Se deficiencies in pregnant women might have adverse effects on the developing fetus, particularly the nervous system. A case-control study of pregnant women found a positive correlation between low Se levels and the occurrence of neural tube defects [22]. However, it is important to note that defects in the nervous system development are affected by multiple other factors and not solely by Se deficiencies. Se deficiencies during pregnancy were also found to cause oxidative stress partly resulting in miscarriages, pre-term deliveries, intrauterine growth retardations, preeclampsia, thyroid dysfunctions, gestational diabetes, and cholestasis [9,23]. Therefore, it is important to maintain optimal Se levels during pregnancy by increasing Se intake to meet the increased demand. 

### 3.2. Diabetes Mellitus

The relationship between Se and diabetes was investigated in multiple studies with contradicting results [18]. Those studies were conducted initially following the assumption that Se is likely to exert protective effects against diabetes due to its antioxidant actions. In support of this assumption, Se was found to delay the onset and progression of diabetes. Se was also found to act as an insulin mimetic when in the form of selenite. Moreover, several in vitro and in vivo studies have suggested that Se plays an important role in the regulation of glucose homeostasis [18,24]. Human studies have also investigated the impact of Se on diabetes yet with conflicting conclusions. Both low and high levels of Se have been shown to be associated with a risk of diabetes [2]. The role of Se in diabetes was assessed in few randomized control trials (RCT). The first large RCT was the Nutritional Prevention of Cancer (NPC) trial which aimed at assessing the effect of 200 µg of daily Se supplementation on reducing the risk of skin cancer compared to placebo [25]. A secondary analysis of the data after a follow-up of 7 years suggested a statistically significant increase in the incidence of type 2 diabetes among those who received Se supplementation compared to placebo [26]. In contrast, the Se and Vitamin E Cancer Prevention Trial (SELECT) found no statistically significant increase in the incidence of type 2 diabetes after Se supplementation [27]. The third trial is the Se and Celecoxib (Sel/Cel) Trial. The intervention was a randomized, placebo controlled trial of Se (200 µg/day) and cyclooxygenase 2 selective inhibitor, celecoxib (400 mg, once per day), alone or in combination, for the prevention of colorectal adenoma in men and women. After exclusion of participants who had type 2 diabetes prior to the trial, 1640 participants were assessed for the presence of type 2 diabetes during the follow-up period. Authors found that 31 participants randomized to Se and 25 randomly assigned placebo were diagnosed with type 2 diabetes; however, despite a higher number of cases in the Se arm, the difference was not statistically significant. Interestingly, the study showed a statistically significant increase in the risk of type 2 diabetes in elderly individuals, which suggests that with old age supplementation with Se may increase the risk of type 2 diabetes [28]. These findings mirror the controversy on whether higher Se concentrations could increase the risk of type 2 diabetes or not. 

Observational studies have also shown a non-linear association between Se and type 2 diabetes. A recent, comprehensive meta-analysis that was conducted in 2019 among 20 observational studies concluded that high Se levels are significantly associated with type 2 diabetes (pooled odds ratios, 1.88; 95% confidence interval, 1.44 to 2.45). However, significant heterogeneity was observed within different studies and the analysis of the funnel plot revealed significant publication bias. Subgroup analysis was also conducted according to the method of Se measurement in each study. Significant association was shown among studies that used blood, diet, and urine as specimens for Se level detection but not in studies that used nails as a sample for Se measurement. Sensitivity analysis was conducted in addition to the trim and fill analysis to account for the heterogeneity and the publication bias, yet the results were consistent upon all adjustments [18]. Another non-linear dose–response analysis confirmed that both levels below and above the physiological range are potential risk factors for diabetes which is consistent with other review articles that suggested a U-shaped relationship between Se and diabetes. Positive associations were detected among patients with low Se levels (<100 μg/l) and patients with high Se levels (>130 μg/l) [29].

### 3.3. Cardiovascular Disease (CVD)

Se as an antioxidant was assumed to play an essential role in protecting against atherosclerotic events and CVD [30]. Se deficiency was directly linked to Keshan disease, an endemic cardiomyopathy that is found in regions where Se intake is very low [31]. Multiple studies were carried out to assess the impact of Se on CVD risk. Both low and high levels of Se were found to adversely affect heart function [2]. In Eastern USA, a post hoc analysis of the NPC trial was conducted with a follow-up of 7.6 years to study the effect of Se supplementation on the prevention of CVD. The incidence of myocardial infarction, total cerebrovascular accidents and CVD were assessed, and the results revealed that there is no overall benefit of supplementing 200 µg/day of Se to prevent CVD [26]. The SELECT trial did not also find any significant effect of Se on overall cardiovascular events [27]. A meta-analysis of RCTs showed that oral Se did not have an overall effect on cardiovascular events regardless of Se formulation or dose [32]. Observational studies have shown conflicting conclusions. A meta-analysis of observational studies concluded a nonlinear, U-shaped relationship, between Se levels and CVD risk. The suggested range of Se concentrations that results in significant beneficial effects against CVD is from 55 to 145 μg/l [32]. 

### 3.4. Cancer

The role of Se in cancer prevention was extensively studied in various cancer types. Se exerts its action through the antioxidant activity of selenoproteins in addition to several other mechanisms [33]. However, some laboratory studies suggested that Se can promote malignant cell transformation and progression [34]. Therefore, whether Se promotes or prevents cancer is not fully clear. Various RCTs investigated Se supplementation for cancer prevention. The first RCT is the NPC trial that aimed at assessing the influence of Se on the development of non-melanoma skin cancer. The NPC trial concluded that Se does not exert a chemoprotective effect against the recurrence of non-melanoma skin cancer, yet it significantly reduced the risk for all cancers, and for esophageal, prostate, colorectal, and lung cancers [25]. Another smaller trial among patients who received organ transplants found an unexpected increase in the incidence of non-melanoma skin cancer as a secondary outcome in Se-supplemented group [35]. The SELECT trial was a pivotal study that was carried out in male general population without high risk of prostate cancer to assess the incidence of prostate cancer among Se-supplemented participants compared to placebo. The study found no significant difference in prostate cancer incidence between Se-supplemented and placebo groups. It also detected no difference in overall cancer risk or any other cancer types [27]. Moreover, three recent RCTs evaluated Se impact on participants with high risk for prostate cancer and all showed no beneficial effect of Se on the incidence of cancer [36,37,38]. Additionally, a trial among women with high risk of breast cancer due to mutations to breast cancer type 1 (BRCA1) susceptibility gene showed increased risk of all cancers and primary breast cancer in Se-supplemented arm compared to placebo [39]. Observational studies have also assessed Se effect on cancer risk with strongly conflicting results. A recent meta-analysis of observational cohort studies showed an overall lower risk of cancer among participants with the highest baseline exposure levels, yet the included studies are subject to a substantial risk of bias [40]. 

## 4. Regional Variability of Se Status: A Focus on the Situation in the Middle Eastern and North Africa (MENA) Region

**Figure 2:**
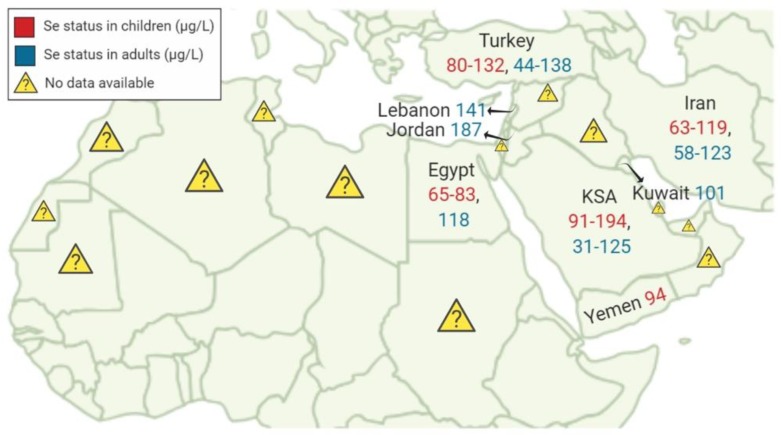
Available data on Se status in children and adults among countries of the MENA region.

The status of Se across the MENA region is widely variable with some countries deficient, some over sufficient and some sufficient in addition to several countries with unknown Se status. This variability was apparent even between provinces from the same country. These tremendous variations may be linked to the varying concentration of Se in the soil were food consumed is gown. We summarized in this review the available evidence on Se status and intake among countries of the MENA region. It is important to note that most of the studies that assessed Se concentrations in biological samples were case-control studies with small sample sizes that cannot be generalized to the whole population. Few studies addressed Se status in a small number of countries with total paucity of information in many other countries of the MENA region. Available evidence on Se status among countries of the MENA region are summarized in Figure 2. 

### 4.1. Turkey

A study that analyzed Se concentrations in colostrum, transitional and mature breast milk among healthy lactating women concluded that Se content is below the international reference range set at 18.5 μg/l among all different samples throughout the lactation period [41]. Moreover, analysis was extended to dairy milk products in Turkey collected from different cities and its findings revealed that goat milk had the highest content followed by sheep milk and finally cow milk had the lowest Se content [41]. A more recent study included all dairy products in the analysis and found that concentrations varied based on the type of food with butter and cheese having higher concentrations; however, other products such as ice cream, milk, and yogurt had almost no Se content [42]. The estimated daily intake of Se among children was reported as 30-40 μg/day [43]. However, with regards to Se concentrations in blood, multiple case-control studies were conducted among different healthy and patient populations including infants, children, adults, pregnant women, and postmenopausal women. Other studies also used samples from erythrocytes and semen to assess Se concentrations [44,45]. Overall, values were widely variable ranging from 50–138 μg/l in healthy populations [43,44,45,46,47,48,49,50,51,52,53,54,55]. 

### 4.2. Jordan

A survey of groundwater was conducted to assess the levels of Se in multiple aquifers in Amman Zarqa Basin. Se levels were various among different aquifers ranging from 0.09 to 742 μg/l with an average of 24 μg/l, which exceeds WHO recommended threshold for drinking water [56]. A later case-control study of colorectal cancer patients assessed the dietary intake of Se which was found to be 59.26 ± 8.91 µg/day for controls. This value is higher than the RDA (55–70 µg/day) [57]. On the other hand, Se concentrations were analyzed in two studies using blood and hair samples [58,59]. Blood concentrations in non-smokers were found to be around 187 μg/l which is relatively high [58]. 

### 4.3. Iran

Multiple studies were conducted in Iran among various populations, children, adults, and elderly [60,61,62]. Se intake was found to be adequate in children and adults [60,61], whereas in postmenopausal women, Se intake was significantly lower than the RDA (55 μg/day) [62]. Se levels in rice samples from Iran were assessed and they were found to be relatively high [63]. Se status in Iran was assessed in various studies in healthy and patient populations. Blood concentrations ranged from 58–123 μg/l in children, adults and pregnant women [64,65,66,67,68,69,70,71,72,73,74,75,76,77].

### 4.4. Kingdom of Saudi Arabia (KSA)

A study was conducted to assess the content of Se in wheat grains grown in KSA. This study reported highly variable concentrations ranging from 8 to 293 μg/kg [78]. Other recent studies that quantified the intake of Se estimated the intake to be 75–121.65 μg/day and 93 μg/day in the regions of Jeddah and Riyadh, respectively [79,80]. Moreover, surveys of infant milk formulas, breast milk and cow’s milk were conducted to determine Se intake in infant/children populations. The findings of those surveys showed that infant milk formulas contained adequate amounts of Se whereas some breast-fed infants might have lower than recommended Se intakes [81,82]. On the other hand, Se status was assessed in multiple case-control studies in KSA. Those studies were done using various samples; serum, urine, toenails, whole blood, umbilical cord blood, and placental tissue [5,80,83,84,85,86,87,88,89,90,91]. Blood concentrations varied widely from as low as 32 μg/l to 195 μg/l [5,80,85,86,87,88,90,91]. 

### 4.5. Egypt

Se intake among healthy children in Egypt was found to be 8.3 ± 2.3 mg/day [92]. Case-control studies in Egypt were mainly done on children, and reported Se concentrations ranging from 65–83 μg/l [93,94,95]. Studies have also assessed Se concentrations in neonates and their mothers and the concentrations were found to be 86 μg/l and 118 μg/l, respectively [96]. 

### 4.6. Qatar

In Qatar, no direct studies were conducted to assess the intake of Se among Qatari population. However, according to Qatar General Electricity & Water Corporation (Kahramaa) drinking water quality requirements report, Se is not expected to be present in Qatar’s water system [97]. This indicates that the Qatari population is not expected to have any Se intake from drinking water sources. Additionally, a market basket survey of Se in rice imports in Qatar, the primary staple dish, concluded that for Qatari citizens, rice compensates for more than 100% of RNI Se (30 μg/day) regardless of the gender or the type of rice consumed. However, for non-Qatari expatriates (over 80% of the population), with a much lower rice consumption, the percentages were variable according to gender and type of rice consumed, yet they were all below 100% of RNI Se [98]. Further analysis included rice-based infant cereals in Qatar which was found to provide around 63% of RNI Se based on the recommended daily serving [98]. This can conclude that rice consumption in Qatar is a significant contributor to the daily intake of Se. However, Se status in Qatar was not assessed in any of the few studies available.

## 5. Conclusions

Se plays a very important role in health and hence Se status and intake are very important to be known for a specific population as the levels of Se are highly variable among different populations and regions. Studies conducted in the MENA region to assess Se status and intake are very limited and most of those available were designed as case-control protocols with small sample sizes. For those countries with data available such as Iran, Turkey, and KSA, more powered, rigorous, and well-designed studies should be conducted to assess Se status among different populations in that specific country or regions that share more or less similar lifestyle and climate such as the six countries of the Gulf Cooperation Council (GCC). Se intake should also be assessed using food surveys and through surveying Se content in staple food (e.g., rice) especially that most of food products consumed in a large number of countries of the MENA region are imported from various parts of the world which may have variable Se content in the soil. Moreover, for many countries within the MENA region, including Qatar and most North-African countries, data about Se status is totally absent. This warrants the need for rigorous and well-constructed studies to determine Se intake and status among the general population and specific populations, such as children and pregnant women, in those countries. Furthermore, studying the relationship between Se status and the incidence of chronic diseases, such as diabetes and CVD in the MENA region would help in devising novel preventive approaches for these disorders particularly prevalent in many countries of this region.

## Figures and Tables

**Table 1:** Functions and significance of selenoproteins

Selenoprotein	Symbol	Function and Significance
Glutathione peroxidase 1	GPX1	Reduces cellular H_2_O_2_. Overexpression of GPX1 increases risk of diabetes.
Glutathione peroxidase 2	GPX2	Reduces peroxide in gut. GPX1/GPX2 double-knockout mice develop intestinal cancer; one allele of GPX2 added back confers protection.
Glutathione peroxidase 3	GPX3	Reduces peroxide in blood. Important for cardiovascular protection, perhaps through modulation of nitric oxide levels; antioxidant in thyroid gland.
Glutathione peroxidase 4	GPX4	Reduces phospholipid peroxide. Genetic deletion is embryonic lethal; GPX4 acts as crucial antioxidant, and sensor of oxidative stress and pro-apoptotic signals; required for spermatozoa function.
Glutathione peroxidase 6	GPX6	Importance unknown.
Iodothyronine deiodinase 1	DIO1	Important for systemic active thyroid hormone levels.
Iodothyronine deiodinase 2	DIO2	Important for local active thyroid hormone levels.
Iodothyronine deiodinase 3	DIO3	Inactivates thyroid hormone.
Thioredoxin reductase 1	TXNRD1	Reduction of cytosolic thioredoxin. Genetic deletion is embryonic lethal.
Thioredoxin reductase 2	TXNRD2	Reduction of mitochondrial thioredoxin. Genetic deletion is embryonic lethal.
Thioredoxin-glutathione reductase	TXNRD3	Reduction of thioredoxin, testes-specific expression.
Selenoprotein H	SELENOH	Involved in transcription. Essential for cell survival and antioxidant defense in Drosophila.
Selenoprotein I	SELENOI	Possibly involved in phospholipid biosynthesis.
Selenoprotein K	SELENOK	Involved in calcium flux in immune cells and endoplasmic reticulum (ER)-associated degradation.
Selenoprotein M	SELENOM	Thiol-disulfide oxidoreductase localized in the ER. Possibly involved in protein folding.
Selenoprotein F	SELENOF	Thiol-disulfide oxidoreductase localized in the ER. Possibly involved in protein folding.
Selenoprotein N	SELENON	Potential role in early muscle development. Mutations lead to multiminicore disease and other myopathies.
Selenoprotein O	SELENOO	Potential redox function, but importance remains unknown.
Selenoprotein P	SELENOP	Se transport to tissues particularly brain and testis. It also functions as intracellular antioxidant in phagocytes. Knockout leads to neurological problems and male sterility.
Methionine-R-sulfoxide reductase 1	MSRB1	Functions as a methionine sulfoxide reductase and MSRB1 knockouts show mild damage to oxidative insult.
Selenoprotein S	SELENOS	Transmembrane protein found in plasma membrane and ER. Reduces ER stress.
Selenoprotein T	SELENOT	ER protein involved in calcium mobilization.
Selenoprotein V	SELENOV	Testes-specific expression, potential role in male reproduction.
Selenoprotein W	SELENOW	Potential antioxidant role, perhaps important in muscle growth.
Selenophosphate synthetase 2	SEPHS2	Involved in the synthesis of all selenoproteins.
